# Stem cell therapy for the treatment of psychiatric disorders: a real hope for the next decades

**DOI:** 10.3389/fpsyt.2024.1492415

**Published:** 2025-01-07

**Authors:** Rosa Villanueva

**Affiliations:** Servicio de Psiquiatría y Salud Mental, Hospital Universitario La Paz, Hospital La Paz Institute for Health Research (IdiPAZ), Universidad Autónoma de Madrid, Madrid, Spain

**Keywords:** bipolar disorder, schizophrenia, major depression, treatment-resistant depression, autism spectrum disorder

## Abstract

In this review, it is evaluated the progress in the application of stem cell therapy to ameliorate the symptoms of bipolar disorder, major depression, schizophrenia, and autism. These disorders are highly prevalent in clinical medicine and are responsible for high levels of psychosocial disability among patients. All of them share common biomedical features, such as complex and variable genetic substrates, significant susceptibility to environmental changes, and insufficient knowledge of their pathogenesis. In addition, the responsiveness of patients to pharmacological treatment is heterogeneous, and in some cases, no treatment is available. Therefore, the development of stem cell-based regenerative medicine and its possible combination with emerging therapeutic approaches that promote neural plasticity are expected to advance neuropsychiatry in the next few decades.

## 1 Introduction

The identification of stem cells in adult organisms, including humans, combined with the design of methodological approaches to transform adult cells into pluripotent stem cells (iPSCs) (1), has opened a new field of extraordinary importance in medicine for the progress of diagnosis and biological characterization of distinct pathologies and for the treatment of diseases for which we previously had no pharmacological tools (2).

Physiologically, stem cells are undifferentiated cells that self-renew and eventually differentiate into specific cell lineages (3). In adults, stem cells are present in many tissues and organs and often occupy niches that maintain their “stemness” (4). Under normal conditions, these cells are attracted by their neighboring tissues to replace damaged cells, ensuring tissue homeostasis (5). However, their regenerative potential is insufficient to overcome a pathological situation. In clinical practice, autologous or allogeneic stem cells can be isolated from the bone marrow, adipose tissue, or umbilical cord blood and expanded *in vitro* to obtain a sufficient number of cells required for cell therapy. A hallmark in modern medicine was the development of iPSCs by genetic reprogramming adult somatic cells *in vitro* (6), thus overcoming the limitations in obtaining the number of cells required for therapeutic procedures.

**Figure 1:**
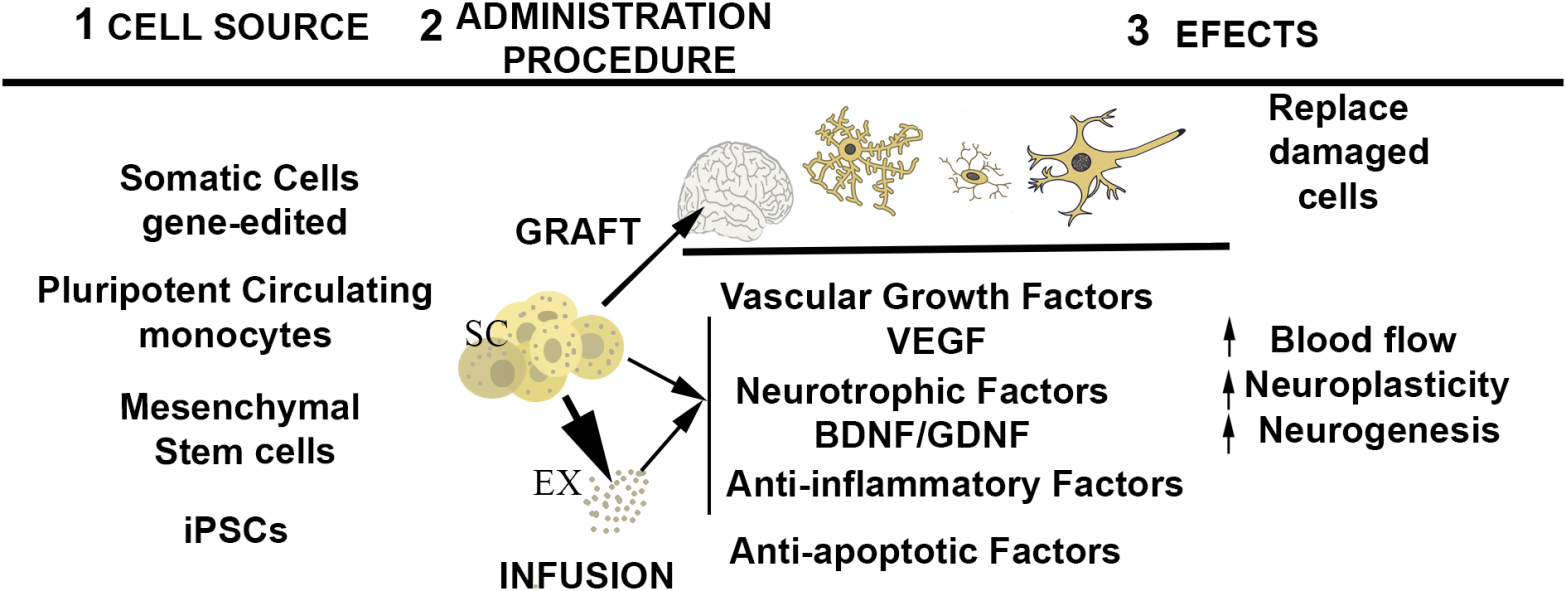
Schematic representation of the central events of stem cell therapy. From left to right: 1) potential source of stem cells; 2) administration procedure; 3) expected effects of stem cell administration. ST, stem cells ready for infusion or engraftment, EX, exosomes released by stem cells.

The beneficial effects expected from the utilization of stem cell-based medicine are not only due to the direct replacement of damaged tissues (7) but also to the release of paracrine factors and extracellular vesicles (exosomes) that interact with the target tissues (8, 9) (**Figure 1**). In fact, it has been found that exosomes delivered by stem cells to the milieu may substitute the employ of the living stem cells. Stem cells, can also be used as carriers for drug delivery to injured organs (2, 10, 11). The latter is favored because of the tropism of stem cells toward damaged tissues (12). In the nervous system, a limitation of the therapeutic use of stem cells is the difficulty of overcoming the blood–brain barrier by noninvasive mechanisms. Advances in the nasal administration of cells or cell products may circumvent the use of invasive approaches (13). The use of experimental animals for the study of psychiatric disorders is limited, but, stem cells grown in different culture assays provide a valuable tool for modeling the pathophysiological substrates of neuropsychiatric disorders. Thus, a complementary and equally important medical application of stem cell technology is the development of tridimensional cultures from iPSCs obtained from patients. These cultures, termed “organoids,” can be directed to differentiate into specific tissue structures and allow monitoring the pathogenic basis of the disease and to test their response to pharmacological treatments, thus predicting their efficacy in a personalized fashion (14, 15).

In the last years important advances have been achieved in obtaining neural-like cells for *in vitro* modelling psychiatric disorders, avoiding reprograming and methodological limitations of iPSC derived from the patients, such as accessibility to obtain the cells, efficiency and time required for differentiation, economical cost, and, of note, the maintenance of the epigenetic signature of the patient cells that is loss during reprograming process (16). Among the sources of neural cells, it can be emphasized the olfactory neuroepithelium (17–19), but somatic cells (i.e. fibroblasts) transfected with lineage-determining transcription factors via gene editing that not require reprogramming (20), and mesenchymal stem cells, or circulating pluripotent monocytes transdifferentiated by growing in conditioned culture medium provide also satisfactory results (21).

The purpose of this review is to summarize the current status of the application of stem cell technology to ameliorate the symptomatology of bipolar disorder (BD), major depression (MD), schizophrenia (SQZ), and Autism Spectrum Disorder (ASD). These disorders are most prevalent in clinical medicine occupying the second (Depressive disorders), seventeenth (SQZ) and twenty-first (ASD) places in the last rank generated by the “Global Burden of Diseases, Injuries, and Risk Factors Study” (22) that estimates the global disease burden, on the basis of incidence, prevalence, and disability-adjusted life- years (DALYs) for 371 diseases at both country and regional levels. The advancement of stem cell-based regenerative medicine and its possible combination with emerging therapeutic approaches that promote neural plasticity opens a promising panorama for the advancement of neuropsychiatry (23).

## 2 Neuropsychiatric disorders

### 2.1 Bipolar disorder

BD is a highly prevalent disorder, affecting more than 1% of the world population. It is characterized by alternating manic and major depressive episodes, and usually develops in young individuals. Despite the influence of environmental factors on the evolution of BD, its genetic origin is accepted. However, rather than single mutations, complex and variable genetic modifications have been identified in individuals and families (24). Since many decades, clinical treatment to reduce manic episodes of BD patients relays in the administration of mood stabilizers (lithium, valproate, and lamotrigine) although their action mechanism was not fully understood (25) and a considerable number of patients fail to respond to the medication.

Studies using stem cell technology have paid much attention to decipher the molecular and cellular basis of the disorder and to unravel the action mechanism of mood stabilizers, including the variability in the responsiveness to lithium treatment (26–30). Organoids made with iPSC obtained from BD patients, revealed important molecular and structural alterations respect to control subjects that are ameliorated by addition of lithium to the culture medium, and, importantly, this *in vitro* assay replicates the responsiveness of the patient to treatment (26, 29–31). This property constitutes a major step to the development of personalized medicine in clinical practice.

The use of organoid technology provided important advances in the understanding of BD pathogenesis (26). Functional and transcriptional studies have revealed increased expression and response to pro-inflammatory cytokines (IL-6) in astrocytes derived from patient iPSCs (31), supporting a potential therapeutic effect of anti-inflammatory treatments (32), to inhibit neuroinflammation (27). Furthermore, genes regulating neuronal differentiation, and plasticity, such as the Wnt/β-catenin signaling pathway appear altered in BD-patients (33, 34), and the influence of Wnt signaling inhibitors in the vitro assay opened new approaches for the treatment of the disorder (35). The microRNA miR34 is another target of lithium altered in BD-derived organoids. It has been proposed that its detection in plasma might be a biomarker, to distinguish lithium responding and non-responding patients (36).

In contrast with the advances obtained in the pathogenesis and conventional pharmacological treatment of the disorder via stem-cell technology, clinical trials for cell therapy in BD are currently in their early stages (37). Several research centers have registered clinical trials using stem cells in combination with conventional mood stabilizers; however, to our knowledge, these results have not yet been published.

### 2.2 Major depressive disorder

MDD is a multifactorial psychiatric disorder characterized by persistent sadness, low self-esteem, and a loss of interest in for environmental stimuli, accompanied by various cognitive and physical symptoms. MDD has the highest prevalence in Western countries and is responsible for social disabilities and suicidal behaviors (38). The pathogenesis of depression involves an intricate combination of genetic, environmental, and neurobiological factors. Genetic predisposition plays a significant role, with multiple genes contributing to vulnerability to depression, particularly when combined with environmental triggers, such as chronic stress or trauma (39–42).

Neurobiologically, MDD is associated with the dysregulation of key neurotransmitter systems, particularly serotonin, norepinephrine, and dopamine, which are crucial for mood regulation (43). Recently, the glutamatergic system has been implicated (44). Additional pathogenic alterations include dysregulation of the hypothalamic-pituitary-adrenal axis, alterations in the gut microbiota (45, 46), microglia and astrocyte modifications in response to inflammatory stimuli (47, 48), and neuroplasticity deficits (49). Collectively, these factors contribute to the onset, progression, and recurrence of depressive episodes.

Selective serotonin reuptake inhibitors are the first-line treatment for MDD. However, at least 30% of the patients are resistant to treatment (treatment-resistant depression; TRD) owing to unknown neurobiological mechanisms. Considering the severity of the disease, this limitation makes it necessary to develop effective treatments for TRD. Combining distinct therapies and the development of novel treatments such as ketamine, psychodynamic drugs, or transcranial magnetic stimulation have provided hopeful results (50, 51).

Stem cell therapy might address neurobiological deficits in TRD (52); however, most data are largely derived from preclinical animal studies (53). Treatment with umbilical cord stem cells (54) or adipose-derived mesenchymal stem cells (55) has revealed positive results in mouse models of depression-like behavior. Studies devoted to improving cardiac ischemia using umbilical cord stem cells have shown that this treatment ameliorated depression-like behavior caused by ischemia (56). In these studies, the effects of cell therapy were associated with the immunomodulatory and anti-inflammatory properties of stem cells (57). A complementary beneficial effect proposed for stem cell therapy is the stimulation of endogenous neurogenesis and neuroplasticity (58–60) or the protection of neurons from induced apoptosis (61). Implantation of encapsulated mesenchymal stem cells into the lateral ventricle of rats ameliorated depression-like behavior, promoting neurogenesis in the subventricular zone and dentate gyrus of the hippocampus (60). Remarkably, the trophic influence of stem cells may be substituted with exosomes obtained from cultured bone marrow mesenchymal stem cells (61). In humans, a preliminary pilot study in 16 female TRD patients subjected to 4 intravenous injections of umbilical cord stem cells (250 x10^6^ cells and 1-week intervals) showed an ameliorative effect on cognitive impairment, and helped overcome resistance to conventional treatment (62). Together, those findings suggest that new treatments combining stem cells and drugs with active neuroplastic activity, such as ketamine (63), may provide more efficient therapies for TRD (50). However, the use of stem cell therapy in humans still requires further animal testing (64). It must be evaluated if cell infusion is enough to induce beneficial effects, or if treatments require neural engraftment. It is also required to know if anti-depressant drugs modify the therapeutic efficiency of stem cells, or the importance of the stage of differentiation of the stem cells. At the present, I have identified three active clinical assays registered in the platform Clintrial.gov designed to evaluating the safety, efficacy and tolerability of stem cells and exosomes for the treatment of depression (NCT02675556: Phase I trial that investigates the administration of allogeneic MSCs in TDR patients that lack posted results; NCT03522545: Phase I trial, at recruiting stage, that evaluates the safety and efficacy of allogeneic bone marrow-derived MSCs in BD patients; NCT03265808: Phase I/II trial, lacking posted results, that investigates the administration of allogeneic MSCs in patients with alcohol use disorder and major depression).

### 2.3 Schizophrenia

SCZ is a severe and prevalent chronic behavioral and cognitive disorder characterized by broad and heterogeneous clinical symptoms, including hallucinations, delusions, cognitive impairment, and social withdrawal. Its pathophysiology is complex and involves critical interactions between genetic and environmental factors (65). SCZ is highly heritable (66), and dozens of genomic loci have been associated with this disorder (67). Current treatment options include psychosocial interventions and antipsychotic drugs that often cause undesired side effects; most importantly, these treatments remain largely ineffective in almost one-third of patients (68).

The characterization of pathogenic alterations in neural progenitors from SCZ patients is currently an intense area of research (19, 69–71). Organoid, and other *in vitro* technologies, have provided substantial advances in our knowledge of the molecular basis of SCZ (72). According to these studies, a core physio-pathogenic feature of SCZ is an unbalanced specification of excitatory and inhibitory neurons together with mitochondrial alterations that increase oxidative stress (73). Dysregulation of neuronal differentiation may be secondary to deficiencies in Wnt signaling (72), which, as mentioned above, is also a feature shared by BD (34). Remarkably, it has been detected a dysregulation of circulating stem cells displaying neural lineage markers in SCZ patients experiencing their first psychotic episode that might serve as a biological marker of SCZ (74).

Currently, monoamine-based antipsychotic drugs are the conventional pharmacological treatments for SCZ; however, they have several adverse effects and limited effectiveness on negative and cognitive symptoms (75). Hence, developing innovative therapeutic approaches in modern psychiatry is challenging. Advances in regenerative medicine have promoted growing interest in stem cells as a potential novel treatment for SCZ (76). Preclinical studies have demonstrated the beneficial effects of a single intravascular infusion of human umbilical cord stem cells on SCZ-related behaviors induced by amphetamine administration in mice (77). Considering that the blood–brain barrier prevents cells from reaching the neuronal centers and the regulation of IL-10, this effect was explained by the immunomodulatory influence of stem cells. This interpretation is supported by recent experiments demonstrating the alleviation of neuroinflammation and synaptic damage repair by regulating the activity of microglia in a maternal immune activation rodent model (78). Moreover, stem cells may also confer beneficial effects via local neuroprotective mechanisms, as shown in both intracranial transplantation of mesenchymal stem cells (MSCs) and intranasal delivery of MSC-derived extracellular vesicles, which alleviated behavioral and biochemical deficits in mouse models of drug-induced SCZ (79, 80). Human studies are still limited to a pilot study, without control patients, designed to assess the safety of the procedure and to analyze cortical activity in a cohort of 15 patients with SCZ (F20.6 in the International Classification of Diseases-10) who received 4 intravenous injections of umbilical cord blood cells (250 × 10^6^ at 2-week intervals). Of note, three months after the last injection, fMRI analysis showed increased cerebral cortical activity in both the anterior and posterior components of the verbal working memory loop, which was interpreted as due to induced neuronal plasticity (76).

### 2.4 Autism spectrum disorder

ASD is a heterogeneous neurodevelopmental condition that manifests in early childhood and persists throughout life. Symptoms of ASD include social and communication impairments along with repetitive, stereotyped behaviors that are frequently associated with other neuropsychiatric and non-psychiatric diseases (81). Genetic and environmental factors are implicated in the development of ASD. Genetic risk factors are complex and involve variants ranging from point mutations to large copy number variants that are either inherited or spontaneous (82). Dysregulation of the gut microbiota has been proposed as an environmental factor in the disease (83). Causal heterogeneity, together with variability in clinical phenotypes, prevents the establishment of a precise basis for the disorder (84), but its pathogenesis includes immune dysregulation, mitochondrial dysfunction, and increased oxidative stress (85).

Despite intense efforts devoted to basic and clinical research (86–90), the design of effective pharmacological treatments for the core symptoms of ASD remains elusive (91). Considering that neuroinflammation, neuronal cell damage, and oxidative stress area central events in ASD, cell therapies using stem cells of different origins have been explored. Many preclinical studies in animal models (92, 93) and human clinical trials (94, 95) have been conducted over the last decade. Except for the absence of therapeutic efficacy reported by Dawson et al. (96) that might be explained by the therapeutic protocol employed in the trial (97), the administration of umbilical cord blood cells (98–102), or autologous bone marrow stem cells (94, 103), using distinct protocols (intrathecal/intravenous) improved autism symptoms without major adverse events. In some studies, clinical improvement was considered low (104); however, in other trials, the treatment modified the spectral characteristics of the electroencephalogram (99) and improved the structural brain connectivity detectable by white matter tractography (101, 105).

Overall, the positive findings described above are largely explained by the immunoregulatory properties of stem cells (101, 105); however, whether this is the only mode of action has been questioned (85).

## 3 Conclusions and ethical considerations

The stem cell technology, via *in vitro* modeling, is an active area of research that, in spite of some limitations (16), is providing great advances in the understanding the pathophysiology of neuropsychiatric disorders (14). Here, I have summarized the efforts of researchers to add mental disorders to the list of pathologies that could benefit from stem cell therapy. In theory, stem cell therapy may benefit psychiatric disorders by two different mechanisms: firstly, being integrated into the target neural regions to substitute deficient neurons; and, secondly, employing the cells as source of factors that ameliorate the structural deficits observed in the mental disorders (**Figure 1**). The first approach, requires the grafting of progenitors into the target neural tissues. This approach is still at initial preclinical stage. The infusion of stem cells with therapeutic purpose is at a more advanced stage of research. A number of published pilot studies evaluated the effectiveness of stem cell therapy to ameliorate symptomatology in MDD, SCZ, and ASD, and all of them concluded that, as observed in other pathologies, the methodology is safe, and devoid of serious adverse events. A critical aspect for the use of stem cell treatments in psychiatry is the importance of providing adequate information on risks/benefits to obtain adequate consent from the patient or their legal guardians, especially, in patients who lack decision-making capacity.

Based on the reported observations, most studies conclude that the effects of stem cell therapy have to do with immune regulation, protecting astroglia and microglia from neuroinflammatory damage, which in turn improves neuronal functions. Additionally, there is a yet uncharacterized trophic effect that promotes neuronal plasticity. Importantly, some reports suggest that cell therapy could be replaced with exosomes derived from stem cells (9, 106). To date, we do not have viable protocols as to determining the most appropriate cell type to use or the method of administration but the data reviewed here are encouraging and suggest that the regenerative treatments alone or in cooperation with other therapeutic approaches could offer solutions in the coming decades to resolve neuropsychiatric pathologies for which we currently have no effective therapies.
